# Comparison of the efficacy of erector spinae plane block performed with different concentrations of bupivacaine on postoperative analgesia after mastectomy surgery: ramdomized, prospective, double blinded trial

**DOI:** 10.1186/s12871-019-0700-3

**Published:** 2019-03-04

**Authors:** Başak Altıparmak, Melike Korkmaz Toker, Ali İhsan Uysal, Semra Gümüş Demirbilek

**Affiliations:** 1 Department of Anesthesiology and Reanimation, Muğla Sıtkı Koçman University, Menteşe Muğla, Turkey; 2 Department of Anesthesiology and Reanimation, Muğla Sıtkı Koçman University Training and Research Hospital, Menteşe Muğla, Turkey

**Keywords:** Analgesia, Erector spinae plane block, Mastectomy, Postoperative pain, Ultrasound

## Abstract

**Background:**

Breast cancer surgery is one of the most common surgeries among the female population. Nearly half of the patients suffer chronic pain following breast cancer surgery, and 24% of them categorizing their pain as moderate to high. In this study, effects of ultrasound-guided erector spinae plane (ESP) block performed using two different concentrations of bupivacaine on postoperative tramadol consumption, pain scores, and intraoperative fentanyl requirements among patients who underwent radical mastectomy surgery were compared.

**Methods:**

This double-blinded, prospective, and randomized study included patients with age ranged 18–70, American Society of Anesthesiologist physical status I–II, and scheduled for unilateral modified radical mastectomy surgery. The patients were randomly allocated into two groups. In group I, ESP block was performed with 0.375% bupivacaine. In group II, ESP block was performed with 0.25% bupivacaine. General anesthesia was induced in both groups according to the standard procedures. When the pain score was ≥4, patients received intravenous (i.v.) 25 mcg fentanyl in the recovery room or 4 mg of morphine in the surgical ward as a rescue analgesia. The main measurements were postoperative tramadol consumption; Numerical Rating Scale (NRS) scores 15, 30, and 60 min and 12 and 24 h postoperatively; and intraoperative fentanyl requirements.

**Results:**

In total, 42 patients (21 patients in each group) were included in the study. The mean tramadol consumption at the postoperative 24th h was 149.52 ± 25.39 mg in group I, and 199.52 ± 32.78 mg in group II (*p* = 0.001). In group I, the NRS scores were significantly lower at every time points compared with those in group II. The mean intraoperative fentanyl requirement was similar in the two groups.

**Conclusion:**

Although ESP block performed with both concentrations of bupivacaine provided effective postoperative analgesia, the higher concentration of bupivacaine significantly reduced postoperative tramadol consumption after radical mastectomy surgery.

**Clinical trial registration:**

The study was registered prospectively with the Australian New Zealand Clinical Trials Registry (trial ID: ACTRN12618001334291at 08/08/2018).

## Background

Globally, breast cancer surgery is one of the most common surgeries among the female population [1]. Nearly half of the patients suffer chronic pain following breast cancer surgery, and 24% of the patients categorize their pain as moderate to high [2]. There are several risk factors for chronic postoperative pain. These include younger age, invasive surgical interventions, and adjuvant radiation therapy following surgery. One of the most important independent risk factors for chronic postoperative pain is a high pain score in the early postoperative period [3].

Ultrasound-guided erector spinae plane block (ESP) is a popular, interfascial regional technique that was initially described for the management of thoracic neuropathic pain [4]. As the erector spinae fascia extends from the nuchal fascia cranially to the sacrum caudally, local anesthetic agents extend through several levels, and the block can be effective over a large area [5]. A case series with three patients reported that ESP block performed with 20 ml of 0.5% ropivacaine reduced postoperative pain scores after laparoscopic cholecystectomy [6]. A randomized clinical trial which evaluated the effectiveness of ESP for the same indication showed that ESP block performed with 20 ml of 0.375% bupivacaine effectively reduced the postoperative pain scores [7]. Although ESP block successfully reduced postoperative opioid consumption in both clinical reports, no studies thus far have investigated the optimum volume or concentration of bupivacaine for ESP block. Thus, in this study, we evaluated the effects of ESP block which was performed by using two different concentrations (0.375 and 0.25%) of bupivacaine in the same volume of solution. Our primary aim was to compare tramadol consumption in the two groups at the end of the postoperative 24th h as assessed by using Student’s *t*-test. Our secondary aim was to compare the intraoperative fentanyl requirements and postoperative pain scores of the groups.

## Methods

The study was designed as a prospective, randomized, double-blinded, clinical trial in accordance with the principles outlined in the Declaration of Helsinki and registered prospectively with the Australian New Zealand Clinical Trials Registry (trial ID: ACTRN12618001334291). The study adheres to CONSORT guidelines. The patients with American Society of Anesthesiologist physical status I–II and aged 18–70 y, were included in the study. All patients were scheduled for an elective unilateral radical mastectomy with axillary lymph node dissection. The patients with coagulation disorders, infection at the injection site, a history of previous mastectomy surgery, chronic analgesic usage, a body mass index ≥35 kg m^2^, and an inability to use a patient-controlled analgesia (PCA) device were excluded.

### Ethics, consent, and permissions

The study was approved by Muğla Sıtkı Koçman University Clinical Research Ethical Committee with the decision number 02–07 and the data were collected in Muğla Sıtkı Koçman University Hospital. Each patient provided written informed consent for the ESP block intervention and participation in the study.

### Patient groups and randomization

In the operating room (OR), standard monitoring with electrocardiography, noninvasive blood pressure, peripheral oxygen saturation, and bi-spectral index monitoring (BIS) was performed in all cases, and the patients’ baseline (0 min) data were recorded. All patients received i.v. 0.05 mg/kg^− 1^ (with a maximum dose of 3 mg) of midazolam for sedation. The patients were then randomly allocated into two groups. A computerized randomization table was created by a researcher who was not involved in the study. For each randomized patient, an anesthesiologist (MKT) took the corresponding sealed envelope from a folder indicating the treatment assigned to the patient and prepared a 0.25% bupivacaine or 0.375% bupivacaine solution in two identical 20 ml syringes. In addition, a 10 ml syringe of isotonic saline was prepared for the hydro-location technique. The first anesthesiologist then passed the labeled syringes to a second anesthesiologist (BA) who was also blinded to the group allocations.

### Block interventions

The patients were placed in a sitting position for the ESP block interventions. ESP block interventions were performed similar to a previous study [8]. In the first group (group I), the second anesthesiologist (BA) located the ultrasound probe in a longitudinal orientation at the level of the T4 spinous process, then placed the probe 2–3 cm laterally from the midline. After the identification of the T4 transverse process and overlying trapezius, rhomboideus, and erector spinae muscles, the targeted injection site was anesthetized with 3–4 ml of 2% lidocaine. An 80 mm 21-gauge block needle was inserted using the in-plane technique following the same injection point in the cranial to caudal direction until the tip contacted to the T4 transverse process. When the correct needle tip position was confirmed by hydro-location with 3–5 ml of isotonic saline solution, the anesthesiologist injected 20 ml of 0.375% bupivacaine deep into the erector spinae muscle. In the second group (group II), the same block procedure was repeated with 20 ml of 0.25% bupivacaine solution. Following block procedures, patients were placed in a supine position.

### Anesthesia protocol

The anesthesiologists (BA and MKT) induced general anesthesia with 2–3 mg/kg^− 1^ of i.v. propofol, 1 mcg/kg^− 1^ of i.v. fentanyl and 0.6 mg/kg^− 1^ of i.v. rocuronium bromide. Following endotracheal intubation, all patients received 4–6% end-tidal desflurane in 2 L of a 40% O_2_ and 60% N_2_O mixture for maintenance of anesthesia. The minimum alveolar concentration of desflurane was regulated to maintain a BIS value between 40 and 60. All patients received 4 mg of i.v. ondansetron and 8 mg of dexamethasone for postoperative nausea and vomiting (PONV) prophylaxis. Intraoperatively, i.v. 75 mg of dexketoprofen trometamol was applied in both groups. The OR anesthesiologists applied i.v. fentanyl (0.5 mcg kg^− 1^) when the hemodynamic parameters of the patients increased more than 20% of the baseline measurements. The inhaled gases were ended at the end of the skin closure and i.v. atropine 0.01 mg/kg^− 1^ and i.v. neostigmine 0.05 mg/kg^− 1^ were administered to reverse the neuromuscular blockage. The patients were transferred to the recovery room following extubation, In the recovery room, another blinded anesthesiologist (AIU) followed patients with 11-point Numerical Rating Scale (NRS) to evaluate postoperative pain. The NRS ranges from “0” (no pain) to 10 (the worst pain imaginable). The patients rated their pain intensity during coughing, and their NRS scores were recorded at the postoperative 15th and 30th min. All patients received a patient-controlled analgesia (PCA) device on their arrival in the recovery room. The PCA device was set to administer an i.v. 10 mg bolus dose of tramadol with a 20-min lock-time and no basal infusion. Patients with NRS scores ≥4 received i.v. 10 mg tramadol via the PCA device. In cases where NRS scores remained ≥4 at the 30th min, patients received 25 mcg of i.v. fentanyl as rescue analgesia. Patients who required i.v. fentanyl stayed in the recovery room for 60 min. All other patients were sent to the surgical ward at the postoperative 30th min.

Subsequent pain assessments during coughing were carried in the surgical ward at the postoperative first, second, 12th, and 24th h using the NRS. In cases where a patient had received i.v. tramadol three times in the last hour and still had a pain score ≥ 4, i.v. morphine (4 mg) was administered as rescue analgesia in the ward. At the same time as the pain assessment, the patients were questioned about nausea and vomiting. The severity of nausea was assessed by the patients themselves on a 4-point scale (none, mild, moderate, and severe). If the patients had moderate or severe nausea or vomiting, they received 10 mg of i.v. metoclopromide. The incidence of severe nausea and vomiting were also noted in the nurse care records.

The primary outcome measure of the study was total tramadol consumption 24 h after the operation. The secondary outcome measures were the NRS scores at the different assessment times and intraoperative fentanyl requirements. In addition, postoperative fentanyl requirements, postoperative morphine requirements, intraoperative hemodynamic parameters, the incidence of PONV, and complications related to the ESP block interventions were recorded.

### Evaluation of the data

The sample size of the study was calculated using the G*Power program (v3.1.9) based on a preliminary study with 15 patients in each group. At least a 20% reduction in total tramadol consumption in the postoperative 24th h was accepted as clinically significant. The mean tramadol consumption was 142.6 ± 28.34 mg in group I, and it was 196.6 ± 29.38 mg in group II. Assuming an α error = 0.01 (two-tailed), with a power of 0.90, at least 18 participants were needed per group. Considering the possible dropouts, 21 patients were decided to include in each group.

### Statistical analysis

The statistical analysis was conducted using Number Cruncher Statistical System 2007 software (Kaysville, UT, USA). Descriptive variables were assessed using statistical methods (mean, median, minimum-maximum, standard deviation, and ratio), and the Student’s *t*-test was used for the comparison of parametric variables with a normal distribution between the two groups. For the comparison of parametric variables without a normal distribution, the Mann–Whitney *U* test was used. Pearson chi-square test and Fisher’s exact test were used for comparison of qualitative data. Three separate generalized linear mixed models were conducted to analyze the effects of group and time on NRS, HR and MAP. NRS, HR and MAP were considered as dependents variables group was introduced as between-subjects variable and time as within-subjects variable. A *p* value < 0.05 was accepted as significant.

## Results

**Fig. 1:**
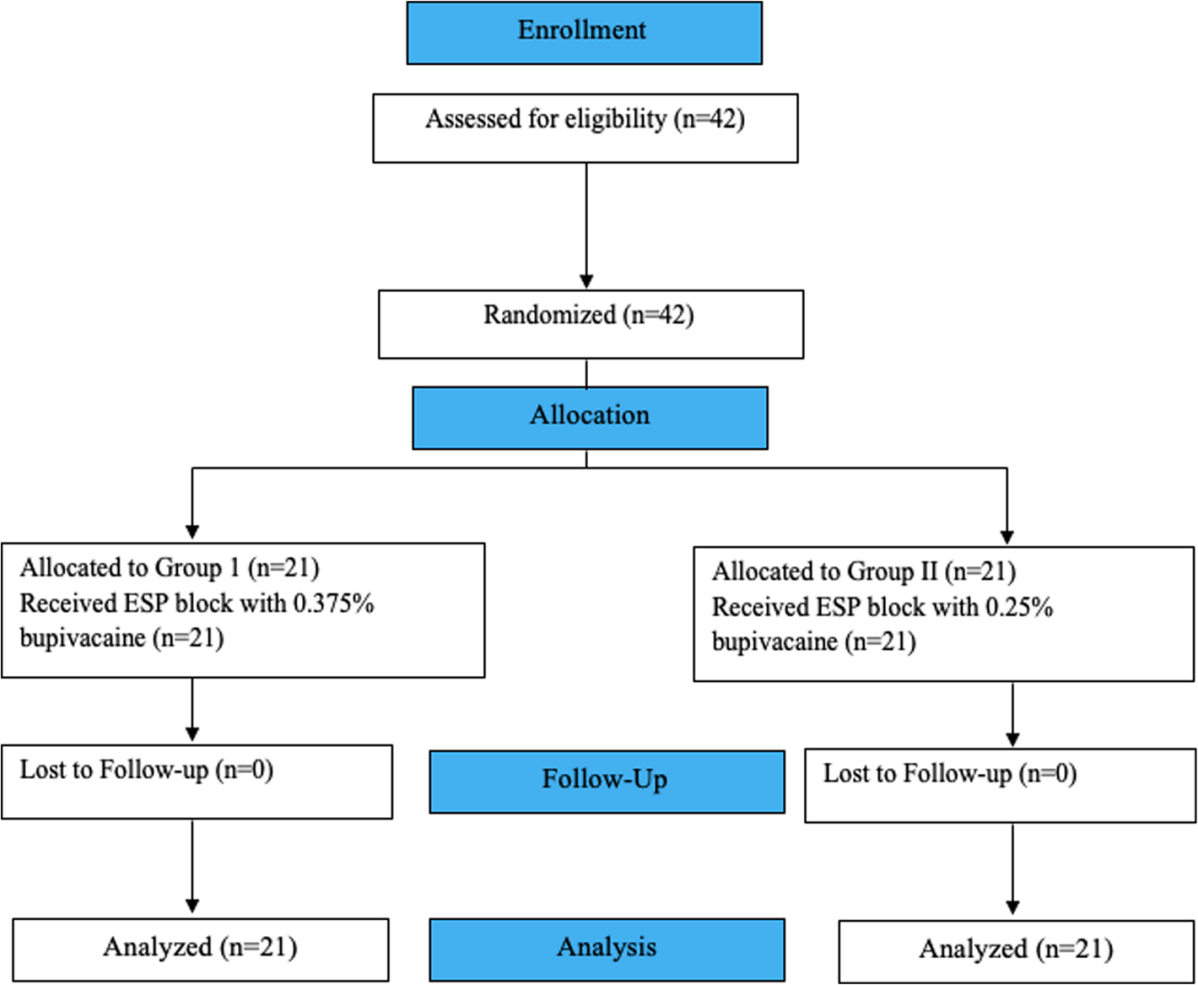
Flowchart of the study

In total, 42 patients were enrolled into the study and there was no dropout (Fig. 1). The enrolled patients’ ages were ranged between 38 to 70 y, with a mean age of 54.62 ± 9.64 y. The demographic variables of the patients in the two groups were similar to each other (Table 1).

**Table 1:** The descriptive variables of the patients in each group

	Group I (*n* = 21)	Group II (*n* = 21)	*p*
Age (year)	53,33 ± 10,33	55,90 ± 8,95	0,394
Weight (kg)	72,33 ± 8,64	75,00 ± 8,81	0,328
Height (cm)	161,62 ± 3,90	160,67 ± 3,53	0,412
BMI (kg/m^2^)	27,71 ± 3,01	28,93 ± 3,16	0,206
Operation time (min)	119,52 ± 15,08	122,38 ± 11,79	0,498
ASA status I	12 (57,1%)	8 (38,1%)	0,217
ASA status II	9 (42,9%)	13 (61,9%)	

*BMI*: Body mass index, *ASA*: American society of Anesthesiologists

Age, weight, height, BMI and operation time values are expressed as mean ± SD

ASA status is expressed as *n* (%)

Although intraoperative fentanyl requirements of the two groups were similar, there were significant differences in the postoperative tramadol consumption and rescue analgesic requirements. The mean tramadol consumption at the postoperative 24th h was significantly higher in group I compare to the group II (*p* = 0.001). Similarly, the postoperative rescue analgesia requirements in the surgical ward were significantly higher in group I as compared with those in group II (*p* = 0.030). None of the patients required fentanyl as rescue analgesia in the recovery room (Table 2).

**Table 2:** Intraoperative fentanyl need and postoperative analgesic requirements

	Group I (*n* = 21)	Group II (*n* = 21)	*p*
Intraop fentanyl (mcg)	90,86 ± 27,22	95,24 ± 21,82	0,289
Postop tramadol (mg)	149,52 ± 25,39	199,52 ± 32,78	0,001
Rescue analgesic in RR (fentanyl) (*n*)	0	0	1.000
Rescue analgesic in SW (morphine) (*n*)	8 (38.1%)	15 (71.4%)	**0,030**

*RR*: Recovery room, *SW*: surgical ward

Intraoperative fentanyl requirement and postoperative tramadol consumption values are expressed as mean ± SD

Rescue analgesic requirements in RR and SW are expressed as *n* (%)

**Fig. 2:**
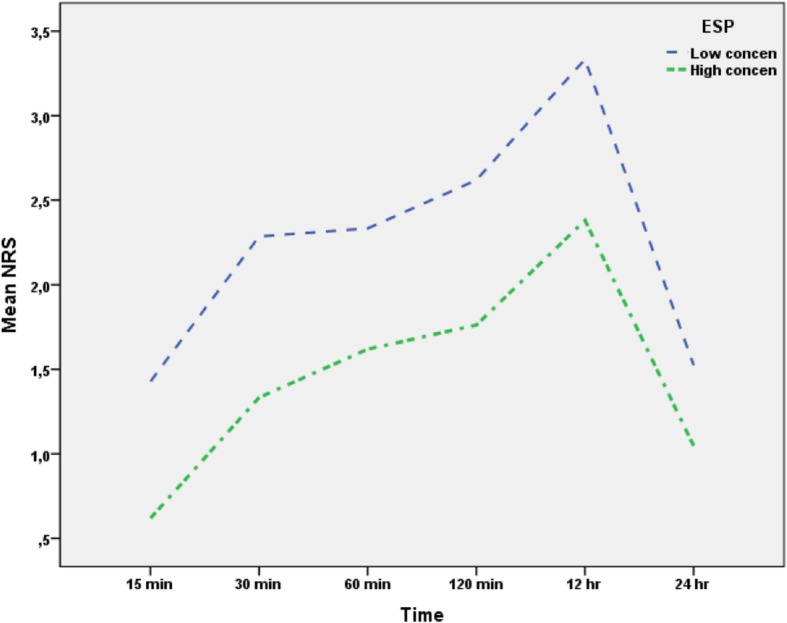
NRS scores of the patients at different time-points of follow-up

Three separate generalized linear mixed models were conducted to analyze the effects of group and time on NRS, HR and MAP. In the first model NRS was the dependent variable where group was introduced as between-subjects variable and time as within-subjects variable. Model, main effect of time and time were statistically significant (F:35.415, *p* < 0.001, F:32.998, *p* < 0.001, F:59.880, p < 0.001, respectively). The effect of interaction term was not statistically significant, implying that changes in NRS values were similar in both groups (Table 3). Although interaction term was not statistically significant, all post-hoc pairwise comparisons were shown in Fig. 2. The median NRS score of patients were statistically high in Group II at the postoperative 15th, 30th, 60th, 120th minutes, 12th h and 24th h (*p* = 0.001, *p* = 0.001, *p* = 0.003, *p* < 0.001, *p* = 0.001, *p*:0.049, respectively). However, the median NRS scores remained < 3 in the postoperative first 24 h. In Group I, significant increases in the NRS scores were detected at the postoperative 30th, 60th, and 120th min and 12th h as compared with the 15th min scores (*p* < 0.001, *p* < 0.001, *p* < 0.001, *p* < 0.001, respectively). Similarly, significant increases in the NRS scores were detected at the postoperative 30th, 60th, and 120th min and 12th h as compared with the 15th min scores in Group II (*p* < 0.001, *p* < 0.001, *p* < 0.001, *p* < 0.001, respectively). However, the median NRS scores were < 4 in both groups in the postoperative first 24 h and the difference in the median NRS scores of groups was < 2 in the first 12 h.

**Table 3:** Effects of group and time on HR and NRS values

	HR		MAP		NRS	
	F	p	F	p	F	p
Model	22.820	**< 0.001**	8.535	**< 0.001**	35.415	**< 0.001**
Group	1.117	0.298	0.519	0.479	32.998	**< 0.001**
Time	17.462	**< 0.001**	4.913	**0.002**	59.880	**< 0.001**
Group x Time	1.189	0.363	1.143	0.385	0.920	0.475

In the second model, HR was the dependent variable where group was introduced as between-subjects variable and time as within-subjects variable. Model and main effect of time were statistically significant (F:22.820, *p* < 0.001, F:17.462, *p* < 0.001, respectively). The effects of main effect of group and the interaction of group and time was not statistically significant, implying that changes in HR values were similar in both groups (Table 3). Although interaction term was not statistically significant, all post-hoc pairwise comparisons were reported in Table 4.

**Table 4:** The evaluation of heart rate changes among groups

HR	Group I (*n* = 21) mean ± sd		Group II (*n* = 21) mean ± sd		p
0.min	80,52 ± 11,11		82.95 ± 9.30		0.435
5.min	79,29 ± 14,83		78.19 ± 12.11		0.790
10.min	85,38 ± 9,89		86.10 ± 9.80		0.810
20.min	81,14 ± 8,03		85.62 ± 8.05		0.071
30.min	77,05 ± 8,60		81.48 ± 8.94		0.102
40.min	75,52 ± 7,03		79.29 ± 8.16		0.110
50.min	73,24 ± 8,04		78.00 ± 7.60		0.051
60.min	74,67 ± 8,56		76.43 ± 8.03		0.484
70.min	75,10 ± 8,14		77.33 ± 8.58		0.378
80.min	77,48 ± 10,03		78.52 ± 7.72		0.699
90.min	78,95 ± 10,20		79.81 ± 8.34		0.761
	Difference (mean ± sd)	p	Difference (mean ± sd)	p	‡p
0.min - 5.min	−4.76 ± 8.93	**0.014**	−1.24 ± 10.39	0.580	0.300
0.min - 10.min	3.14 ± 9.15	0.128	4.86 ± 7.35	0.002	0.520
0.min - 20.min	2.67 ± 6.52	0.078	0.62 ± 9.58	0.763	0.562
0.min - 30.min	−1.48 ± 7.67	0.395	−3.48 ± 9.89	0.110	0.632
0.min - 40.min	−3.67 ± 6.63	0.018	−5.00 ± 10.93	0.044	0.880
0.min - 50.min	−4.95 ± 8.09	0.008	−7.29 ± 9.26	0.001	0.465
0.min - 60.min	−6.52 ± 10.34	0.008	−5.86 ± 10.57	0.014	0.940
0.min - 70.min	−5.62 ± 10.28	0.020	−5.43 ± 10.29	0.019	0.930
0.min - 80.min	−4.43 ± 10.12	0.051	−3.05 ± 12.07	0.244	0.970
0.min - 90.min	−3.14 ± 11.26	0.199	−1.57 ± 11.64	0.530	0.930

Generalized linear mixed model with LSD post-hoc tests

‡Comparison of differences between low and high content groups

In the third model MAP was the dependent variable where group was introduced as between-subjects variable and time as within-subjects variable. Model and main effect of time were statistically significant (F:8.535, *p* < 0.001, F:4.913, *p*:0.002, respectively). Main effect of group variable and effect of the interaction of group and time was not statistically significant, implying that changes in MAP values were similar in both groups (Table 3). Although interaction term was not statistically significant, all post-hoc pairwise comparisons were reported in Table 5.

**Table 5:** The evaluation of mean arterial pressure changes among groups

Time-points	Group I (*n* = 21)		Group II (*n* = 21)		p
0.min	102,48 ± 7,75		104.29 ± 6.81		0.430
5.min	97,71 ± 11,21		98.48 ± 8.67		0.802
10.min	103,10 ± 9,25		106.19 ± 7.03		0.251
20.min	102,29 ± 10,80		104.67 ± 5.99		0.374
30.min	99,67 ± 10,11		102.48 ± 7.81		0.309
40.min	99,05 ± 7,00		99.76 ± 9.35		0.776
50.min	98,29 ± 8,50		98.81 ± 9.37		0.847
60.min	98,19 ± 9,85		97.05 ± 8.21		0.681
70.min	97,95 ± 9,13		97.33 ± 6.81		0.801
80.min	98,00 ± 9,10		100.24 ± 7.89		0.389
90.min	100,33 ± 10,35		101.00 ± 7.48		0.808
postHoc	Difference (mean ± SD)	p	Difference (mean ± SD)	p	
0.min - 5.min	−4,76 ± 11,84 (−9)	0.069	−5,81 ± 10,93 (−8)	**0.015**	0.632
0.min - 10.min	0,62 ± 9,98 (3)	0.771	1,9 ± 5,97 (3)	0.150	0.960
0.min - 20.min	−0,19 ± 10,2 (1)	0.932	0,38 ± 6,82 (1)	0.794	0.960
0.min - 30.min	−2,81 ± 11,01 (−2)	0.267	−1,81 ± 7,93 (−1)	0.310	0.597
0.min - 40.min	−3,43 ± 9,72 (−5)	0.129	−4,52 ± 10,04 (− 5)	0.069	0.641
0.min - 50.min	−4,19 ± 12,54 (−6)	0.183	−5,48 ± 10,04 (− 5)	**0.046**	0.743
0.min - 60.min	−4,29 ± 13,04 (−3)	0.240	−7,24 ± 9,8 (−10)	**0.005**	0.257
0.min - 70.min	−4,52 ± 11,49 (−1)	0.221	−6,95 ± 7,93 (−8)	**0.001**	0.227
0.min - 80.min	−4,48 ± 12,29 (−2)	0.183	−4,05 ± 9,24 (−7)	0.051	0.420
0.min - 90.min	−2,14 ± 13,98 (−1)	0.517	−3,29 ± 8,27 (− 3)	0.069	0.529

Values are expressed as mean ± SD

Six patients (28.5%) in group I and seven patients (33.3%) in group II had moderate to severe nausea in the postoperative period. However, none of the patients had vomiting. None of the patients had any other complications in the first postoperative 24 h. In the postoperative period, two patients in each group had pain at the injection site as a complication of ESP block.

## Discussion

In the current study, ultrasound-guided ESP block performed using two concentrations of bupivacaine (0.25 and 0.375%) provided effective analgesia after radical mastectomy surgery. However, the ESP block at the higher concentration of bupivacaine reduced postoperative tramadol consumption more significantly than the lower concentration of bupivacaine. In addition, the NRS scores of group I were significantly lower than group II. The intraoperative fentanyl requirements of the patients in the two groups were similar.

ESP block has been used for postoperative analgesia of several painful conditions since 2016. In these studies, the researchers used different concentrations of bupivacaine, ropivacaine, and lidocaine during block procedures [9–12]. Veiga et al. [13] performed ESP block using 20 ml of 0.5% levobupivacaine for postoperative analgesia in a patient who underwent unilateral mastectomy surgery. We evaluated the effect of ESP block using the same volume and the same patient group. However, we performed ESP block with 0.25 and 0.375% concentrations of bupivacaine. In the present study, even the lower concentration of local anesthetic agent provided effective analgesia. Similarly, two recent studies evaluated the effect of ESP block by using 20 ml of 0.25% bupivacaine. These studies reported that ESP block provided effective postoperative analgesia after unilateral mastectomy surgery [10, 13].

In the current literature, although different local anesthetic agents and different concentrations of these agents were reported to be effective for postoperative analgesia, there are no randomized controlled trials to evaluate effects of ESP block performed with different concentrations of a local anesthetic agent. Some authors recommend the use of a larger volume and lower concentration of local anesthetic solution to avoid systemic local anesthetic toxicity. Moreover, a larger volume of local anesthetic solution tended to cover more segments for postoperative analgesia [14]. On the other hand, a higher local anesthetic concentration is known to allow better diffusion into the paravertebral space, thereby result in more effective nerve block [15]. Ueshima et al. [16] reported two cases of failed ESP block to provide complete analgesia after radical mastectomy surgery. They used 25 ml of 0.25% bupivacaine during the ESP block intervention and detected an incomplete analgesic effect on the T2–T6 intercostal nerves 20 min later. The authors asserted that the ESP block performed with 25 ml of 0.25% bupivacaine was insufficient to affect the anterior branches of the T2-T5 spinal nerves. Similarly, Ivanusic et al. [17] showed that ESP block performed with 20 ml of 0.25% methylene blue dyed only the posterior and lateral branches of the thoracic nerve. However, origins of ventral and dorsal branches of the thoracic spine were not involved in their cadaveric study. According to these data, the applied volume or the concentration of the local anesthetic agent was insufficient to affect the ventral branches. We cannot be sure whether an ESP block performed with a higher concentration of local anesthetic solution would pass through the paravertebral space to provide a more effective nerve block, or not.

Therefore, in the current study we decided to compare two different concentrations of the same local anesthetic agent in the same volume of solution. Although both concentrations provided effective analgesia in the postoperative period, the ESP block performed using the higher concentration of bupivacaine reduced postoperative opioid consumption more significantly.

Besides the above advantages, ESP block performed using a higher concentration of local anesthetic agent may have disadvantages as well. First, bupivacaine overdose and systemic toxicity should be considered in surgeries, such as laparoscopic cholecystectomy, that require bilateral ESP block for effective postoperative analgesia. In the present study, we performed unilateral ESP block and used a total dose of 75 mg of bupivacaine in all the patients. Therefore, there was no potential risk of bupivacaine overdose. Second, patients with low body weights have an increased risk of local anesthetic toxicity, especially when bilateral block is performed using a higher concentration of solution. Third, the median difference in the NRS scores of groups was statistically significant at almost all time-points in the present study, however, the difference may not be considered clinically significant. The difference in the NRS scores of the groups was < 1.2 throughout the first postoperative 24 h. To improve the safety of regional anesthesia, the target should be the minimum dose of local anesthetic agent capable of providing maximum effectiveness [18].

In both groups, the NRS scores tended to differ after postoperative 30th min and reach their maximum values at the postoperative 12th h. This is more likely related to the pharmacokinetic properties of bupivacaine. The pharmacokinetics of local anesthetic agents depend on the rate of systemic uptake, distribution, and elimination of the drug from the body. The vascularity of the injection site is the primary factor that determines the absorption of the drug. In addition, bupivacaine increases the capillary blood flow when used at a concentration of 0.25–0.5%. The distribution of local anesthetics also depends on whether the uptake is by less perfused organs or highly perfused organs. Finally, the elimination half-life is the most important indicator of how soon another dose can be applied safely [18]. Kopacz et al. [19] assessed the pharmacokinetic properties of 0.25% ropivacaine and 0.25% bupivacaine for intercostal nerve blocks in healthy male volunteers. They reported that pinprick anesthesia was detected within 5 min and the maximum level of sensorial blockade was observed 2 h following the block intervention. They observed a significant decrease in sensorial blockade after 10 h. These durations were consistent with those in the present study. In our study, the mean operation time was 119 min in group I and 122 min in group II. Therefore, the sensorial blockade was at the maximum level at the end of the operation. The pain scores reached the maximum level at the postoperative 12th h when the sensorial blockade had dissipated.

In a study by Gürkan et al. [11], 32% of patients in an ESP group and 40% of patients in a control group had nausea in the postoperative period. The incidences of nausea in the present study were very similar to those reported by Gürkan et al. [10], with 28.5% of patients in the high ESP group and 33.3% in the low ESP group experiencing postoperative nausea. The most important risk factors for PONV are female sex, a history of motion sickness or PONV, nonsmoking status, and postoperative use of opioids. Postoperative opioid consumption is believed to be the most important reason for PONV, with a reported incidence as high as 79% following opioid use [19]. In the present study, the ESP block performed with a higher concentration of bupivacaine significantly reduced postoperative tramadol consumption and rescue analgesic consumption. Therefore, the most plausible reason for the lower incidence of postoperative nausea in group I is lower postoperative opioid consumption. However, the difference between the groups was not statistically significant. The most likely reason for this result was the intraoperative administration of i.v. ondansetron and dexamethasone for PONV prophylaxis in both groups.

The present study has some limitations. First, the block procedures were performed while the patients were awake, and general anesthesia was induced as soon as the block interventions were completed. Thus, we did not have time to assess the sensory block area and detect potential block failures. However, none of our patients required rescue analgesia in the recovery room. Second, we used 20 ml of local anesthetic solution in both groups. However, some researchers reported to use 25 ml of local anesthetic solutions for ESP block [16, 20]. The optimum volume of local anesthetic solution for ESP block remains unclear.

## Conclusions

Ultrasound-guided ESP block performed with 20 ml of 0.375% bupivacaine reduced postoperative tramadol consumption more significantly than ESP block performed with 20 ml of 0.25% bupivacaine. The potential for systemic overdose should be kept in mind, especially during bilateral block interventions.

## Abbreviations

BIS: Bi-spectral index
ESP: Erector spinae plane block
MAP: Mean arterial pressure
NRS: Numerical Rating Scale
OR: Operating room
PCA: Patient-controlled analgesia
PONV: Postoperative nausea and vomiting
US: Ultrasound
